# The role of adipose tissue dysfunction in hepatic insulin resistance and T2D

**DOI:** 10.1530/JOE-24-0115

**Published:** 2024-08-02

**Authors:** Gencer Sancar, Andreas L Birkenfeld

**Affiliations:** 1 German Center for Diabetes Research, Neuherberg, Germany; 2 Department of Internal Medicine IV, Division of Diabetology, Endocrinology and Nephrology, Eberhard-Karls University of Tübingen, Tübingen, Germany; 3 Institute for Diabetes Research and Metabolic Diseases, Helmholtz Center Munich, Eberhard-Karls University of Tübingen, Tübingen, Germany

**Keywords:** adipose tissue, insulin resistance, MASLD, type 2 diabetes

## Abstract

The root cause of type 2 diabetes (T2D) is insulin resistance (IR), defined by the failure of cells to respond to circulating insulin to maintain lipid and glucose homeostasis. While the causes of whole-body insulin resistance are multifactorial, a major contributing factor is dysregulation of liver and adipose tissue function. Adipose dysfunction, particularly adipose tissue-IR (adipo-IR), plays a crucial role in the development of hepatic insulin resistance and the progression of metabolic dysfunction-associated steatotic liver disease (MASLD) in the context of T2D. In this review, we will focus on molecular mechanisms of hepatic insulin resistance and its association with adipose tissue function. A deeper understanding of the pathophysiological mechanisms of the transition from a healthy state to insulin resistance, impaired glucose tolerance, and T2D may enable us to prevent and intervene in the progression to T2D.

## Introduction

Human physiology is adapted to handle extended periods of food scarcity, followed by short periods of abundance during millions of years of evolution. However, easy access to a high-calorie, low-quality diet together with sedentary lifestyles in today’s world places our bodies under nutrient stress. The liver and white adipose tissue (WAT) can store the energy surplus as relatively inert triacylglycerol (TAG). When the storage limit is exceeded, excessive accumulation of body fat can lead to a range of metabolic abnormalities and diseases, including dyslipidemia, metabolic dysfunction-associated steatotic liver disease (MASLD), β cell dysfunction, prediabetes, and type 2 diabetes (T2D). The common denominator of these metabolic disturbances is insulin resistance, which is a condition in which the body's cells become resistant to the effects of insulin, leading to impaired glucose and lipid homeostasis. Adipose tissue dysfunction plays a crucial role in the development of insulin resistance in T2D. Dysfunctional adipose tissue is characterized by decreased insulin sensitivity, increased inflammation, aberrant lipolysis, and altered adipokine secretion. In people with obesity and even in lean individuals, visceral adiposity and adipose tissue insulin resistance (adipo-IR) are associated with intrahepatic triglyceride accumulation and hepatic insulin resistance (Holt *et al.* 2006, Petersen & Shulman 2018, Stefan 2020, Lee *et al.* 2023).

Hepatic insulin resistance is characterized by impaired insulin signaling in the liver, leading to increased glucose production and decreased glycogen storage, all of which contribute to hyperglycemia. Since the liver plays a significant role in regulating glucose and lipid metabolism, its dysfunction worsens adipocyte function, starting a vicious cycle leading to T2D. Hence, understanding the mechanisms underlying hepatic insulin resistance and its relationship with adipose tissue dysfunction is crucial for developing effective therapeutic interventions to break the cycle and prevent the progression of MASLD and T2D. While the contribution of adipose tissue dysfunction to muscle insulin resistance and β-cell dysfunction plays a key role in T2D progression, it is beyond the scope of this review and has been reviewed elsewhere (Xourafa *et al.* 2023). We will focus on the molecular pathways underlying hepatic insulin resistance and the role of adipose tissue in the progression of T2D. Moreover, we will discuss the efficacy of lifestyle interventions and their mode of action in restoring glucose and lipid homeostasis.

## Hepatic glucose regulation in health and disease

### Molecular pathways underlying hepatic insulin resistance

One of the main signaling cascades of insulin is the phosphoinositide-3-phosphate kinase PI3K/AKT pathway which is pivotal in mediating insulin's effects on anabolic metabolism across organisms. The binding of insulin to the insulin receptor (InsR) activates PI3K through insulin-receptor substrates (IRS), leading to the generation of phosphatidylinositol (3,4,5)-trisphosphate (PIP3). This initiates the recruitment of AKT to the plasma membrane, phosphorylation by phosphoinositide-dependent kinase 1 (PDK1) at Thr-308, and activation of AKT, which is essential for insulin signaling. Furthermore, AKT's full activation requires phosphorylation by the mammalian target of rapamycin complex 2 (mTORC2) at Ser-473. Activated AKT conveys the insulin signal to downstream effectors such as FoxO transcription factors, glycogen synthase kinase 3 (GSK3), and mTORC1/SREBP1 to regulate glycogen, glucose, and lipid synthesis, respectively. Several studies have investigated the signaling pathways involved in the regulation of liver metabolism by insulin and explored the molecular mechanisms underlying hepatic insulin resistance. Interfering with insulin signaling at the receptor level, at PI3K/AKT, and/or at the downstream effectors is suggested to be the underlying cause of hepatic insulin resistance.

### Regulation of insulin receptor activity by PKCε

Insulin resistance in the liver is a complex process that involves multiple molecular pathways. One major hypothesis is that insulin signaling in hepatocytes is impaired at the level of the insulin receptor (InsR) where the signaling starts. Inhibitory phosphorylation of the InsR by protein kinase C-epsilon (PKCε) upon high-fat feeding has been suggested. High-fat feeding increases hepatic diacylglycerol (DAG) levels, resulting in the activation of PKCε. In particular, an increase in hepatic plasma membrane-bound sn1,2-DAG content is associated with hepatic insulin resistance (Birkenfeld & Shulman 2014). The phosphorylation at Thr-1160 by PKCε decreases InsR-Tyr-1162 phosphorylation and insulin receptor kinase activity (Petersen *et al*. 2016). A direct causal relationship between DAG accumulation, PKCε activation, and the development of hepatic insulin resistance has been shown using liver-specific knockdown or overexpression of PKCε (Lyu *et al.* 2020). Acute knockdown of PKCε in the liver after short-term high-fat feeding relieved hepatic insulin resistance in rats, whereas liver-specific overexpression of a constitutively active isoform of PKCε exacerbated hepatic insulin resistance. Consistent with these experiments, liver plasma membrane and lipid droplet-associated sn1,2-DAG content and pInsR-Thr-1160 phosphorylation correlate with insulin resistance in humans (Kumashiro *et al.* 2011, Lyu *et al.* 2020). While InsR-Thr-1160 phosphorylation by PKCε is a crucial mechanism that links increased DAGs to hepatic insulin resistance, this single phosphorylation event is unlikely to fully encapsulate the effect of PKCε on the signaling and physiology of high-fat diet (HFD)-induced hepatic insulin resistance. To identify direct PKCε substrates involved in HFD-induced hepatic insulin resistance, phosphoproteomics and large-scale *in vitro* kinase assays were employed. The substrates of PKCε included RPS6 and IRS1, which suggests crosstalk between PKCε and p70S6K signaling (Brandon *et al.* 2019). While the role of PKCε in insulin resistance is repeatedly shown by independent groups, other signaling pathways could contribute to insulin resistance in the liver, especially upon long-term HFD feeding (Arkan *et al.* 2005).

### Disruption of PI3K/AKT pathway

The dominant role of the hepatic PI3K/AKT pathway in liver insulin signaling and metabolism is well-established. Liver-specific knock-out of IRS1 and IRS2 prevents activation of the pathway in response to insulin, leading to insulin resistance and hyperglycemia, but not hepatic steatosis (Dong *et al.* 2008, Kubota *et al.* 2016). Hepatic PI3K deletion prevents steatosis but results in glucose intolerance and impaired AKT activity (Miyake *et al.* 2002, Taniguchi *et al.* 2006, Chattopadhyay *et al.* 2011). Additionally, phosphatase and tensin homolog (PTEN) counteracts PI3K by dephosphorylating PIP3, and its deletion leads to substantial lipid accumulation in the liver, potentially due to increased AKT2 activity (Horie *et al.* 2004, He *et al.* 2010). AKT2 plays a significant role in metabolic regulation, and its deletion prevents lipid accumulation in the liver. Deletion of both AKT1 and AKT2 is necessary to fully suppress AKT activity in the liver and leads to severe insulin resistance and glucose intolerance (Lu *et al.* 2012, Titchenell *et al.* 2016). Modulation of the PI3K/AKT pathway, either at the transcript level or through post-translation modifications of the signaling, could determine the insulin response of the liver. One culprit that interferes with insulin signaling at the level of downstream signaling is the aberrant deposition of the sphingolipid ceramide. When the hepatocyte's energy needs are met and its storage capacity is full, ceramides accumulate by leading to the coupling of free fatty acids to the sphingoid backbone. As ceramides accumulate, they initially could protect cells from acute increases in fatty acids and enhance triglyceride storage. However, upon prolonged accumulation, ceramide actions can cause insulin resistance and hepatic steatosis. Studies in rodents and humans show that liver insulin resistance and hepatosteatosis are strongly associated with hepatic ceramide content (Luukkonen *et al.* 2016, Apostolopoulou *et al.* 2018, Chaurasia *et al.* 2019). At a molecular level, increased ceramide levels inhibit the insulin signaling cascade at the AKT step. At least two main mechanisms are suggested for the inhibition of AKT activation by ceramides. First, ceramide blocks the translocation of AKT to the plasma membrane via posttranslational modification that involves PKC (Powell *et al.* 2003). The second pathway involves the dephosphorylation of AKT by activating protein phosphatase 2A (PP2A). Inhibition of PP2A, either chemically or genetically, showed resistance to ceramide-induced insulin resistance. Moreover, inhibition of PP2A in hepatocytes increased AKT activity in primary hepatocytes (Galbo *et al.* 2013). Ceramide could also decrease the relay of insulin signaling to AKT via the regulation of IRS1 phosphorylation by activating double-stranded RNA-activated protein kinase (PKR) (Yang *et al.* 2010). While the effects of ceramides on AKT signaling are mainly shown in muscle cells and adipocytes, decreasing ceramide synthesis or increasing its degradation in the liver relieves insulin resistance and decreases hepatic steatosis (Chaurasia *et al.* 2019). In particular, the downregulation of ceramide synthase 6, which produces long-chain C16-ceramides in the liver, protects from obesity-associated insulin resistance and decreases hepatic fat content, suggesting C16-ceramides could be detrimental to liver metabolism (Raichur *et al.* 2014, Turpin *et al.* 2014, Hammerschmidt *et al.* 2019).

### Dysregulation of the effector molecules of insulin action

Insulin signaling regulates gluconeogenesis, glycogen, and lipid synthesis in the liver, which requires orchestrating hundreds of enzymes in a coordinated manner. Hence, dysregulation of the effector enzymes’ activity or their levels could result in blunted insulin action. Insulin can regulate gluconeogenesis via direct and indirect mechanisms in the liver (Lewis *et al.* 2021). FoxO transcription factors play a major role in direct regulation via transcriptional mechanisms. Activation of FoxOs induces the expression of glucose 6-phosphatase (G6PC), phosphoenolpyruvate carboxykinase (PEPCK), and fructose 1,6-bisphosphatase, which are enzymes involved in gluconeogenesis (Matsumoto *et al.* 2007, O-Sullivan *et al.* 2015). Moreover, FoxOs suppress the expression of glucokinase, which results in decreased flux of glucose to glycogen and lipid synthesis (Zhang *et al.* 2006, Dong *et al.* 2008, Haeusler *et al.* 2014). In an insulin-sensitive state, phosphorylation of FoxO via AKT promotes nuclear export resulting in decreased transcription of these gluconeogenic genes. Downregulation of FoxO1 levels or activity in the livers of diabetic mouse models results in lower plasma glucose concentrations and decreased hepatic glucose production (Altomonte *et al.* 2003, Samuel *et al.* 2006) Moreover, while deletion of the insulin receptor in the liver causes hyperglycemia, deletion of FoxO1 in the liver together with the insulin receptor normalizes serum glucose levels, indicating a critical role for hepatic FoxO1 in glucose metabolism. It is important to note that when hepatic insulin signaling is impaired, extrahepatic insulin signaling could still regulate hepatic glucose production, possibly via regulation of substrate flux from adipose tissue (Lu *et al.* 2012, O-Sullivan *et al.* 2015, Perry *et al.* 2015, Titchenell *et al.* 2015, Titchenell *et al.* 2016). Moreover, disruption of hepatic signaling could cause adipo-IR indirectly regulating hepatic glucose production. For example, liver-specific Irs1/Irs2 double-knockout mice show insulin resistance in adipose tissue, which is relieved when hepatic FoxO1 is deleted (Tao *et al.* 2018). At the mechanistic level, insulin resistance observed in adipose tissue of liver-specific Irs1/Irs2 double-knockout mice is driven by, at least in part, increased secretion of a hepatokine called follistatin (Tao *et al.* 2018). In humans, plasma follistatin levels are associated with an increased risk of T2D incidents (Wu *et al.* 2021).

In addition to its role in regulating gluconeogenic gene expression, FoxO1 could integrate insulin signaling with mitochondrial function, which could be important for the regulation of hepatic glucose production at the substrate level (Cheng *et al.* 2009). In humans, FOXO1 is upregulated in insulin-resistant and fatty liver (Valenti *et al.* 2008). Moreover, a newly identified transcription factor, TOX4, which regulates gluconeogenic genes in parallel with FOXO1, is upregulated in the livers of patients with diabetes and MASLD (Wang *et al.* 2022*b*). Hepatic glucokinase levels and activity are lower in patients with T2D, which could contribute to increased hepatic glucose production and decreased glycogen levels in the liver (Clore *et al.* 2000, Haeusler *et al.* 2015). Indeed, mutations of human glucokinase are seen in a specific subtype of type 2 diabetes (GCK-MODY), which results in a chronic, mild increase in blood glucose levels in part due to decreased glucokinase activity in the pancreas and the associated lower insulin release (Ashcroft *et al.* 2023). In addition to gluconeogenesis, the regulation of glycogen synthesis by insulin plays a crucial role in maintaining normal glucose levels. In patients with T2D, insulin-induced synthesis of hepatic glycogen is impaired (Krssak *et al.* 2004). Transgenic mice models that have increased hepatic glycogen synthesis showed enhanced glucose control (O'Doherty *et al.* 2000, Mehta *et al.* 2017, López-Soldado *et al.* 2022). Polymorphisms in genes encoding proteins in glycogen synthesis are associated with increased diabetes risk (Groop *et al.* 1993). These results indicate that increased activity/levels of proteins involved in gluconeogenesis or changes in proteins regulating glycogen synthesis could enhance glucose output from the liver and resist suppression of HGP by insulin.

## Contribution of adipose tissue dysfunction to hepatic insulin resistance

**Figure 1:**
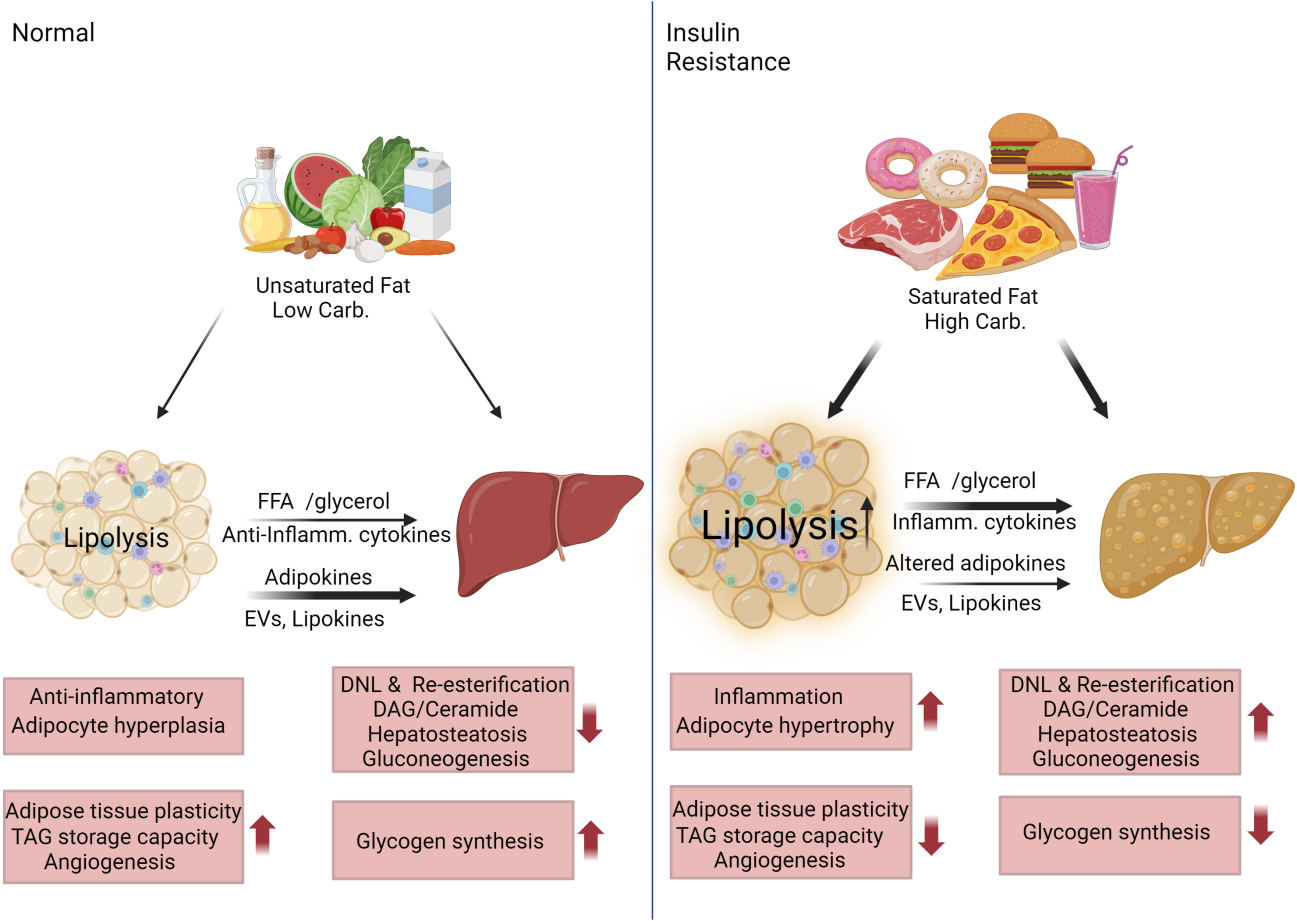
Metabolic changes associated with Western diet-induced insulin resistance. Under normal diet conditions (left panel), adipose tissue has enough capacity to store energy surplus through the generation of new adipocytes (hyperplasia), increased TAG storage, and the formation of new vasculature to support healthy adipose expansion. Adipose tissue is in an anti-inflammatory state, maintaining lipid and glucose homeostasis, while liver function is modulated by adipokines, cytokines, EVs, and lipokines. In contrast, the Western diet overwhelms adipose tissue TAG storage capacity, resulting in adipocyte hypertrophy and inflammation of the adipose tissue. Increased lipolysis, inflammatory cytokines, and altered adipokine release contribute to whole-body insulin resistance and metabolic dysfunction of the liver. In addition, a high saturated fat, high carbohydrate diet enhances lipogenic substrate availability to the liver directly and indirectly. The high glucose and fructose content of the Western diet provides a carbohydrate source for de novo lipogenesis. Moreover, enhanced lipolysis and decreased lipid storage capacity of the dysfunctional adipose tissue provide FFAs and glycerol that increase re-esterification of TAG, exacerbating hepatosteatosis and liver insulin resistance. DAG, diacylglycerol; DNL, de novo lipogenesis; EVs, extracellular vesicles; TAG, triacylglycerol. The figure was created with BioRender.com.

Adipose tissue plays a crucial role in controlling whole-body glucose homeostasis in both normal and diabetic states. White adipose tissue stores excess energy as triglyceride, which can be rapidly hydrolyzed by lipases when needed and transported to other tissues. Adipose tissue also functions as an endocrine organ, secreting a large number of peptide hormones, cytokines, and regulatory lipids that affect energy metabolism in other tissues and behaviors related to feeding. Studies in rodents and humans indicate that the absence of adipose tissue is as detrimental to glucose homeostasis as having excess adipose tissue (Petersen & Shulman 2018, Sakers *et al.* 2022). Upon high energy intake (i.e. high-fat diet), adipose tissue expands via increasing the adipocyte number (hyperplasia) and enlarging existing adipocytes (hypertrophy). However, expansion capacity is limited and dependent on extracellular remodeling followed by the formation of new vasculature (Crewe *et al.* 2017). Under over-nutrition stress, adipocytes release pro-angiogenic and pro-inflammatory factors such as MCP1, TNFα, IL6, ICAM1, VCAM1, etc., which further increase inflammation by activating and recruiting more macrophages (Kratz *et al.* 2014, Sárvári *et al.* 2021). A direct link between adipose tissue inflammation and the role of macrophages was shown in murine models and suggested in humans with obesity (Han *et al.* 2013, Hill *et al.* 2018, Jaitin *et al.* 2019, Vijay *et al.* 2020, Hildreth *et al.* 2021). Increased inflammation, hypoxia associated with inadequate angiogenesis, and lipid overload induce adipocyte insulin resistance. Insulin resistance leads to loss of adipocyte identity and adipose tissue dysfunction (Vishvanath & Gupta 2019, Czech 2020, Roh *et al.* 2020, Markussen & Mandrup 2023). Dysfunctional adipose tissue releases pro-inflammatory factors such as TNF-α, IL-6, and IL-1β, and decreases the release of anti-inflammatory factors such as adiponectin and IL-10, exacerbating hepatic inflammation, insulin resistance, and steatosis (Abenavoli & Peta 2014, Stojsavljević *et al.* 2014). While adipose tissue is an endocrine organ and the main storage place for lipids, adipose dysfunction leads to whole-body insulin resistance via enhanced lipolysis and impaired release of lipokines, adipokines, cytokines, and extracellular vesicles (EVs) (Fig. 1).

### Lipolysis

In a healthy state, adipose lipolysis is tightly controlled by neuronal stimuli and hormones. Pro-lipolytic signals such as noradrenaline activate β-adrenergic signaling in adipose tissue, turning on the cAMP/protein kinase A (PKA) pathway. Phosphorylation of hormone-sensitive lipase (HSL) and perilipin-1 by PKA enhances the recruitment of HSL to lipid droplets and enables the interaction of adipose triglyceride lipase (ATGL) and its co-activator (CGI-58/ABHD5) on the lipid droplets, resulting in increased lipolysis. Other β-adrenergic signaling-independent pro-lipolytic factors include glucocorticoids, natriuretic peptides, and parathyroid hormone, which modulate cAMP/cGMP levels in adipocytes (Braun *et al.* 2018).

One of the most important anti-lipolytic hormones is insulin, which induces the degradation of cAMP in an AKT/PDE3B-dependent manner. Postprandial insulin normally suppresses lipolysis, indirectly suppressing hepatic glucose production via limiting the glycerol and FFA flux to the liver. Adipose dysfunction is often linked to obesity, including an increase in basal rates of lipolysis that contribute to the development of insulin resistance, as well as an impaired fold-response to stimulated lipolysis (Reynisdottir *et al.* 1995, Arner *et al.* 2018). In addition, insulin suppression of adipose lipolysis is also impaired, resulting in enhanced hepatic glucose production (Perry *et al.* 2015). We recently identified that Fibroblast growth factor-1 (FGF1) suppresses lipolysis in a phosphodiesterase 4D (PDE4D)-dependent manner and decreases hepatic glucose production in parallel to insulin (Sancar *et al.* 2022). Over-expression of PDE4D in the adipose tissue of *ob/ob* mice lowered circulating FFAs and glucose levels, directly linking the role of lipolysis to hepatic glucose output. FFAs and glycerol released from adipocytes are taken up by the liver and activate hepatic glucose production via allosteric regulation of pyruvate carboxylase (as acetyl-CoA from fatty acid oxidation (FAO)) or as direct gluconeogenic substrates (as glycerol). In particular, the size of the visceral adipose tissue, which is more lipolytic, more insulin resistant, and drained via the portal vein directly to the liver, strongly correlates with the degree of hepatic insulin resistance and liver fibrosis (Gastaldelli *et al.* 2007, Saponaro *et al.* 2022). Recent single-cell RNAseq experiments identified functionally different cell types and adipocyte subtypes not only in visceral vs subcutaneous but also within the same depot (Vijay *et al.* 2020, Bäckdahl *et al.* 2021, Sárvári *et al.* 2021, Emont *et al.* 2022). Accumulating evidence states that obesity and associated adipo-IR could alter the cell types and function in the adipose tissue, further exacerbating insulin resistance. High-fat feeding and associated obesity lower the lipogenic adipocyte subpopulation and increase the macrophage population within the adipose tissue, increasing the lipolysis and FFA release to other organs (Sárvári *et al.* 2021). Moreover, a locally increased concentration of FFAs potentially promotes a pro-inflammatory macrophage state, further increasing the inflammation and initiating a vicious cycle (Lumeng *et al.* 2007, Kratz *et al.* 2014). In addition, aberrant macrophage differentiation and function in adipose tissue can amplify inflammatory signaling between adipose tissue and the liver in patients with MASH (Boesch *et al.* 2024).

Genetic studies in rodents or human polymorphisms associated with genes involved in lipolysis indicate the direct contribution of unregulated lipolysis to insulin resistance. For example, people carrying frameshift mutations on *PLIN1* (coding for perilipin-1, the most abundant lipid droplet coat protein in adipocytes) show partial lipodystrophy, severe insulin resistance, and T2D (Gandotra *et al.* 2011). This phenotype is associated with increased basal lipolysis. In parallel, *Plin1* knock-out mice show higher lipolysis, increased proinflammatory macrophages, and insulin resistance even in lean mice (Sohn *et al.* 2018). Genetically or diet-induced obese mice show lower Plin1 expression in adipocytes, which could contribute to increased basal lipolysis (Sohn *et al.* 2018). Acute deletion of the insulin receptor specifically in mouse adipocytes resulted in increased lipolysis, insulin resistance, glucose intolerance, hepatosteatosis, and β-cell islet hyperplasia with hyperinsulinemia (Sakaguchi *et al.* 2017).

While *de novo* lipogenesis contributes to fatty liver and associated hepatic insulin resistance, lipid synthesis from FFAs is responsible for 60% of the TAG stored in the liver (Donnelly *et al.* 2005, Smith *et al.* 2020) Moreover, increased lipogenesis observed in patients with fatty liver is driven by the lipogenic substrate availability rather than paradoxical activation by insulin (Ter Horst *et al.* 2021). The data indicate the importance of understanding and potentially targeting adipose lipolysis for relieving hepatic insulin resistance and associated comorbidities.

### Lipokines, adipokines, and extracellular vesicles

Adipose tissue can secrete various bioactive circulating mediators in the form of lipids (lipokines) or peptide hormones (adipokines) that regulate metabolism and behavior (Scheja & Heeren 2019, Tsuji & Tseng 2023). In addition, accumulating evidence indicates adipose tissue-derived extracellular vesicles (EVs), which can carry nucleic acids, proteins, and lipids to distant tissues, are involved in metabolic regulation (Bond *et al.* 2022, Liu *et al.* 2023). The term ‘lipokine’ was introduced after the identification of adipose-derived, palmitoleate (C16:1n7) which decreases hepatic TAG accumulation and increases insulin sensitivity (Cao *et al.* 2008). Circulating palmitoleate was strongly associated with insulin sensitivity in humans, indicating its relevance in humans (Stefan *et al.* 2010, Tricò *et al.* 2020). One of the novel lipid species identified while trying to understand the insulin sensitivity observed in the GLUT4 overexpressing obese mice is branched fatty acid esters of hydroxy fatty acids (FAHFAs) (Yore *et al.* 2014). A palmitic acid-carrying version called palmitic-acid-9-hydroxy-stearic-acid (PAHSA) was studied for its potential anti-diabetic effects. Chronic PAHSA treatment of aged chow-fed or HFD-fed mice enhanced insulin sensitivity and glucose tolerance (Syed *et al.* 2018, Zhou *et al.* 2019). Humans with insulin resistance have lower PAHSA levels in serum and subcutaneous adipose tissue (Yore *et al.* 2014). Exercise induces PAHSAs in adipose tissue, potentially contributing to its benefits (Brezinova *et al.* 2020). At the molecular level, PAHSA’s insulin-sensitizing effects are partly driven by its anti-lipolytic effect in HFD mice (Zhou *et al.* 2019). ATGL, the main TAG lipase in adipose tissue, was identified as the enzyme that synthesizes the FAHFAs (Patel *et al.* 2022). In addition, FAHFA levels were downregulated in whole-body and adipose-specific ATGL-KO mice (Brejchova *et al.* 2021, Patel *et al.* 2022). Another active lipid mediator class is oxidized lipid metabolites derived from polyunsaturated fatty acids (PUFAs). One of the identified molecules is 12,13-diHOME, which enhances fatty acid uptake and catabolism in brown/beige fat and is produced by cold exposure or exercise (Lynes *et al.* 2017, Stanford *et al.* 2018). Studies in humans indicate that 12,13-diHOME plasma levels negatively correlate with insulin resistance and body mass index.

In addition to lipid-based signaling molecules, adipose tissue releases peptide-based adipokines such as adiponectin, leptin, resistin, vaspin, FABP4, RBP4, asprosin, etc., which have been identified over the years, with adiponectin and leptin being the most studied (Zhang *et al.* 1994, Halaas *et al.* 1995, Hu *et al.* 1996, Wang *et al.* 1998, Steppan *et al.* 2001, Yamauchi *et al.* 2001, Hida *et al.* 2005, Yang *et al.* 2005, Cao *et al.* 2013, Romere *et al.* 2016). Adipokines carry great potential as biomarkers to track adipose dysfunction, obesity, and insulin resistance. In addition, dysregulation of adipokine release or function has been implicated in the onset of obesity, IR, T2D, and associated complications, indicating they contribute to disease progression rather than only being correlative (Graham *et al.* 2006, Würfel *et al.* 2023). For example, the administration of adiponectin has been shown to lower glucose levels and improve insulin sensitivity in mice (Berg *et al.* 2001). Conversely, mice lacking adiponectin exhibit insulin resistance and are prone to developing diabetes (Maeda *et al.* 2002). Adiponectin achieves these effects through various mechanisms, including increased FAO in muscle and liver, inhibition of hepatic glucose production, and decreasing hepatic ceramides via ceramidase activity of the adiponectin receptors in the liver (Berg *et al.* 2001, Yamauchi *et al.* 2002, Holland *et al.* 2011). Studies have also demonstrated that adiponectin deficiency is associated with insulin resistance and is predictive of hepatic fibrosis in patients with MASLD (Savvidou *et al.* 2009, Nazal *et al.* 2010) that replenishing physiologic doses of adiponectin can reverse insulin resistance (Yamauchi *et al.* 2001, Savvidou *et al.* 2009, Li *et al.* 2020). Moreover, the beneficial effects of PPAR-ɣ agonists on MASH are mainly associated with increased adiponectin release from adipose tissue and decreased visceral to subcutaneous adipose tissue (Skat-Rørdam *et al.* 2019, Gastaldelli *et al.* 2021). Understanding the molecular and physiological actions of adipokines is essential for developing targeted therapeutic agents for adipose dysfunction associated with insulin resistance and T2D.

Recently, EVs originating from adipose tissue have been identified that carry metabolically active lipids, proteins, and nucleic acids (Thomou *et al.* 2017, Bond *et al.* 2022, Wang *et al.* 2022*a*, Xu *et al.* 2023). Mice with a fat-specific knockout of the miRNA-processing enzyme Dicer and individuals with lipodystrophy demonstrate significant reductions in circulating exosomal miRNAs, indicating adipose tissue as a significant source of circulating miRNAs (Thomou *et al.* 2017). The EVs released from subcutaneous adipose tissue or visceral adipose tissue show depot-specific differences and are modulated by metabolic state (Deng *et al.* 2009, Kranendonk *et al.* 2014, Crewe *et al.* 2021, Camino *et al.* 2022, Zhang *et al.* 2023). Injection of EVs from adipose tissue of obese mice to chow-fed healthy mice induced insulin resistance, supporting the link between adipose tissue EVs and insulin signaling (Deng *et al.* 2009, Castaño *et al.* 2018). Analysis of circulating EVs in healthy individuals, people with obesity, or people with obesity and T2D indicated directional change in a variety of miRNAs (Kim *et al.* 2020, Santamaria-Martos *et al.* 2020). In addition to differences in miRNA species, EVs from obese adipose tissue carry lipids and proteins involved in inflammation and insulin resistance compared to lean controls (Kulaj *et al.* 2023, Zhang *et al.* 2023). Together, data suggest that, depending on the adipocyte health and size, adipose-derived EVs represent a unique signature that could be involved in the regulation of metabolism.

The aforementioned signaling molecules released from adipose tissue reflect the contribution of adipose tissue not only as a reservoir of energy in the form of lipids but also as an active endocrine organ. Thus, insulin resistance of adipose tissue directly contributes to whole-body insulin resistance and T2D by modulating the function of other organs and protecting them from lipotoxicity. In addition, paracrine/autocrine signaling to nearby pre-adipocytes/adipocytes or immune cells could modulate insulin signaling in adipose tissue, indirectly affecting whole-body insulin resistance.

### Lifestyle intervention for targeting adipose tissue and insulin resistance

**Figure 2:**
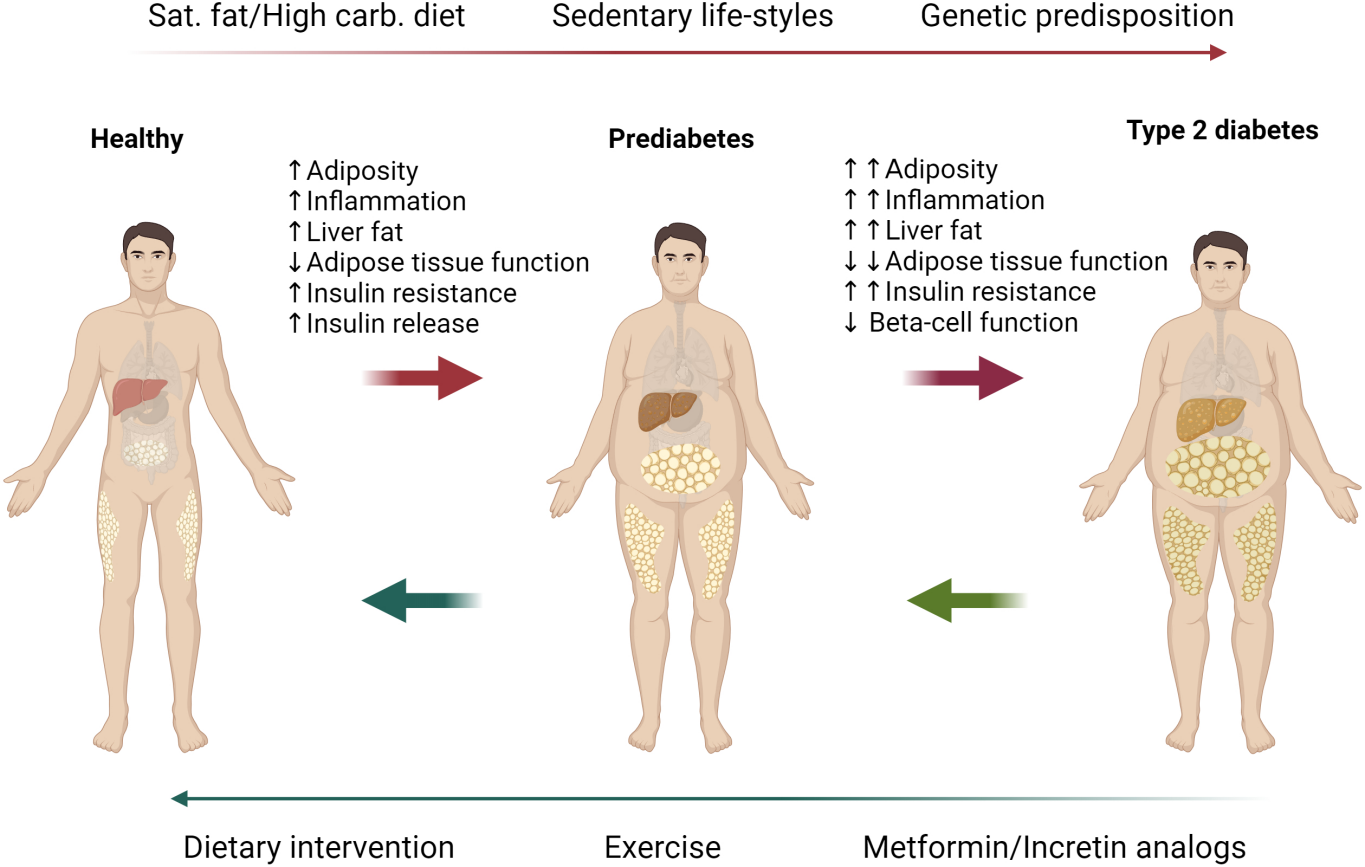
Physiological changes associated with prediabetes/T2D progression and remission. Diet, sedentary lifestyles, and genetic disposition increase the risk of prediabetes and diabetes. Increased visceral adiposity, liver fat, inflammation, and associated insulin resistance impair glucose and lipid metabolism. When not intervened, a further increase in visceral adipose tissue and a decrease in adipose tissue function enhances hepatic steatosis and whole-body insulin resistance. While β-cells respond to insulin resistance by releasing more insulin as a compensatory mechanism during obesity and prediabetes, eventual β-cell failure results in the clinical manifestation of type 2 diabetes (T2D). Through lifestyle interventions such as diet and exercise, individuals with T2D can achieve remission from T2D to prediabetes, and from prediabetes to a healthy state. However, compliance of the patients with these interventions is limited, and medical interventions are still needed. Metformin and newly developed incretin analogs show high potential for managing type 2 diabetes. Yet, there is still a need for novel insulin sensitizers that target the underlying cause of type 2 diabetes, insulin resistance. The figure is created with BioRender.com. Red arrows indicate the transition from healthy to prediabetic to diabetic state with the physiological changes depicted above the arrows. Green arrows indicate remission from diabetes to prediabetes to a healthy state with the help of lifestyle changes and/or with drug treatment (such as metformin and incretin analogs).

The common denominator underlying hepatic insulin resistance is lipid overload in conjunction with dysfunctional adipose tissue. It has been repeatedly shown in animal models and humans that chronic consumption of a high-fat/high-sugar diet results in hepatic steatosis, insulin resistance, and obesity. A high-sugar diet (especially enriched in fructose) has direct effects on the liver, increasing hepatic *de novo* lipogenesis (Eng & Estall 2021, Geidl-Flueck *et al.* 2021, Sigala *et al.* 2021). Decreasing the energy intake by dietary intervention and decreasing the lipid overload has the potential to relieve insulin resistance. The physiological changes from the healthy state to prediabetes and T2D are summarized in Fig. 2. Hence, dietary intervention is the first line of treatment to prevent the progression from pre-diabetes to diabetes or reverse from pre-diabetes/diabetes to a healthy state (Lean *et al.* 2018, Taylor 2019, Iglesies-Grau *et al.* 2023, Sandforth *et al.* 2023, Birkenfeld & Mohan 2023, Jumpertz von Schwartzenberg *et al*. 2024). Weight loss through dietary interventions is the main driver of the remission from (pre)diabetic to a healthy state in most, if not all cases. A very low-calorie diet is particularly effective and needed for T2D remission primarily through weight loss (Kelly *et al.* 2020, American Diabetes Association 2021). Beyond calorie restriction, the macronutrient composition of the diet plays a role in regulating adipose and liver metabolism (El-Agroudy *et al.* 2019). For instance, a low-carbohydrate diet leads to a greater reduction in visceral fat and improves insulin sensitivity compared to a low-fat diet (Samaha *et al.* 2003, Bazzano *et al.* 2014). Additionally, time-restricted eating further impacts visceral adiposity and insulin resistance (Sutton *et al.* 2018, He *et al.* 2022, Sun *et al.* 2024). Together, the data suggest that while a hypocaloric diet is necessary for remission, the type of diet and the time of day the diet is consumed contribute to the full metabolic outcome.

Improvement in insulin sensitivity and reduced visceral adipose tissue are the main drivers for remission from prediabetes to normal glucose regulation (Sandforth *et al.* 2023). Remission from T2D was associated with the recovery of β-cell health and enhanced insulin release (Taylor *et al.* 2018, Zhyzhneuskaya *et al.* 2020). It is of interest to investigate adipose dysfunction and lipolysis in intervention studies for T2D, while increased lipolysis in adipose tissue is associated with lipotoxicity in the islets of the pancreas (Oh *et al.* 2018, Gerst *et al.* 2019). Another lifestyle intervention is regular exercise, which increases energy expenditure, muscle glucose uptake, and FAO in muscle and relieves insulin resistance. Both acute and regular exercise can modulate hepatic gene expression and hepatic and circulating metabolites (Hu *et al.* 2020, Dreher *et al.* 2023). Studies in people with obesity suggested that even without apparent weight loss, regular exercise can decrease hepatic lipid content and visceral adipose tissue (Johnson *et al.* 2009) Moreover, the reversal of insulin resistance in people with obesity was associated with reduced visceral fat (O'Leary *et al.* 2006) While the beneficial effects of dietary intervention and regular exercise are multifactorial, effects on adipose tissue mass and health contribute to relieving insulin resistance. Despite the obvious benefits of lifestyle intervention, applicability and sustainability determine the net positive effect on a large population of people with insulin resistance and T2D. Hence, the search for insulin-sensitizing drugs is still ongoing.

## Declaration of interest

The authors declare that there is no conflict of interest that could be perceived as prejudicing the impartiality of this review.

## Funding

This work was supported by funding from the German Federal Ministry for Education and Research (01GI0925) via the German Center for Diabetes Research (DZD e.V.).

## Data availability

This publication does not contain novel data.

## Author contribution statement

All authors contributed to the writing of this manuscript.
